# Field experiments on solar geoengineering: report of a workshop exploring a representative research portfolio

**DOI:** 10.1098/rsta.2014.0175

**Published:** 2014-12-28

**Authors:** David W. Keith, Riley Duren, Douglas G. MacMartin

**Affiliations:** 1 School of Engineering and Applied Science and Kennedy School of Government, Harvard University, Pierce Hall, 29 Oxford Street, Cambridge, MA 02138, USA; 2 Jet Propulsion Laboratory, California Institute of Technology, Earth Science and Technology Directorate, 4800 Oak Grove Drive, Pasadena, CA 91109, USA; 3 Department of Computing and Mathematical Sciences, California Institute of Technology, 1200 East California Boulevard, Pasadena, CA 91125, USA

**Keywords:** solar geoengineering, solar radiation management, experiment

## Abstract

We summarize a portfolio of possible field experiments on solar radiation management (SRM) and related technologies. The portfolio is intended to support analysis of potential field research related to SRM including discussions about the overall merit and risk of such research as well as mechanisms for governing such research and assessments of observational needs. The proposals were generated with contributions from leading researchers at a workshop held in March 2014 at which the proposals were critically reviewed. The proposed research dealt with three major classes of SRM proposals: marine cloud brightening, stratospheric aerosols and cirrus cloud manipulation. The proposals are summarized here along with an analysis exploring variables such as space and time scale, risk and radiative forcing. Possible gaps, biases and cross-cutting considerations are discussed. Finally, suggestions for plausible next steps in the development of a systematic research programme are presented.

## 1. Introduction

Field research on solar geoengineering is the subject of considerable controversy, with some arguing that no such research should be permitted until international binding governance mechanisms are in place or until relevant avenues of laboratory research are fully exhausted [1,2]. Some of these differences in opinion arise from legitimate and well-founded disagreements about the risks and efficacy of solar geoengineering as well as concerns about the political impediments to making legitimate decisions about its implementation. It is possible, however, that some of the disagreement about field research arises from sharply divergent assumptions about the likely magnitude and risks of initial research projects. Many commentators have assumed that any useful field tests will be large enough to have substantial transboundary effects [3], or even that no meaningful testing of solar radiation management (SRM) is possible without full-scale deployment [4].

In order to sharpen assumptions about likely characteristics of initial field experiments, we convened a workshop in March 2014 to develop a technically credible and broadly representative set of field experiment proposals. This paper reports on the results of the workshop. The motivation is that offering a better description of potential initial field tests will support a healthy debate about the wisdom of such tests while reducing spurious disagreements that arise from the divergent assumptions about the character of field experiments.

The intent here is not to advocate for any particular test, nor to argue for field tests in general. Rather, the goal is to articulate a representative portfolio of possible tests based on scientific merit to support debate about the overall merit and risk of such research as well as mechanisms for governing any such research.

One cannot build an effective regulatory regime without some understanding of what is to be regulated, that is, a quantitative definition of the kind of activities that would occur in the absence of regulation. When we asked workshop participants for proposals for field research to improve understanding of the risks and efficacy of SRM we therefore asked them to assume that such research could be funded and permitted using rules that would be relevant if the experiments were normal atmospheric science unrelated to SRM. Neither the authors nor other workshop participants approached this arguing that SRM research should proceed with no additional governance; the goal is simply to provide a better definition of the scope of potential experiments.

Finally, while the term SRM appears throughout this work, it is used to refer to all methods that directly alter radiative fluxes without modifying long-lived greenhouse gases, specifically including concepts for modifying cirrus clouds with the goal of increasing outgoing long-wave radiation. Note that changes to cirrus clouds will alter both solar and infrared fluxes. Our use of SRM corresponds to radiation management as defined in Boucher *et al.* [5].

## 2. Field research in context: a taxonomy of solar radiation management research

As with the larger atmospheric science research efforts of which it is a part, an SRM research programme would comprise multiple types of research that collectively aimed to improve understanding of relevant Earth system processes and specific technologies (table 1).

**Table 1.** Experiment types. Laboratory testing is included for context; however, the workshop and this analysis were focused primarily on field experiments.

classification	goal	examples
laboratory	understanding efficacy and risks for processes or scales that are well represented by laboratory experiments or models	indoor experiments using climate models, small-scale engineering tests of deployment hardware, laboratory measurement of relevant quantities (e.g. chlorine activation chemistry)
technology development	test hardware and operations needed for deployment	outdoor tests of enabling technologies (e.g. sea spray hardware, hydrosol dispersal or aircraft platforms)
process studies	predictive understanding of the small-scale evolution of physical, chemical and radiative properties in the atmosphere	Controlled release experiments in the atmosphere to understand aerosol/cloud microphysics, chemistry, microscale dynamics, etc. (e.g. aerosol dynamics and O_3_ response to small sulfur release in stratosphere). Passive studies of cirrus clouds and observations of volcanic eruptions, ship tracks or other analogues
scaling tests	bridge gaps across multiple process scales to validate end-to-end model representation	atmospheric experiments to evaluate models across a range of scales including gaps between model domains (e.g. marine cloud brightening test spanning microphysics, large eddy simulation and mesoscale models)
climate response tests	incremental evaluation of climate response to radiative forcing to assess risk and efficacy	slow ramp up over a few decades as sulfur burden in troposphere is reduced; or modulate global radiative forcing over a shorter period

Field experiments, the focus of our study, can be categorized into four types (table 1), three of which—process studies, scaling tests and climate response tests—aim to develop predictive understanding of the efficacy and risks of SRM.

Technology development, the fourth type of field research, supports the needs of field research as well as the potential development and evaluation of hardware and operational methods for solar geoengineering deployment. Technology development, in particular, and the field research programme as a whole comprise both the specific means by which radiative forcing might be altered as well as the development of any new observational systems required to monitor the risk and efficacy of a geoengineering intervention.

Perhaps the most important distinction is between experiments that seek to understand atmospheric *processes* and experiments that aim to understand large-scale *climate response*. Process experiments can involve very small perturbations—the smallest experiments described here involve releases of less than 1 kg of material and produce radiative forcing perturbations that are small compared with that of a single flight of a commercial transport aircraft—yet, these experiments can still provide data that enable improvements in understanding of specific processes. In sharp contrast, experiments that aim to test large-scale climate response may require *global* radiative forcing perturbations of the order of 0.1 W m^−2^ sustained over a decade [6]. As we discuss in §4, quantitative measures of the integrated climate forcing differ by a factor of 100 billion between the largest and smallest proposed experiments.

**Figure 1.**
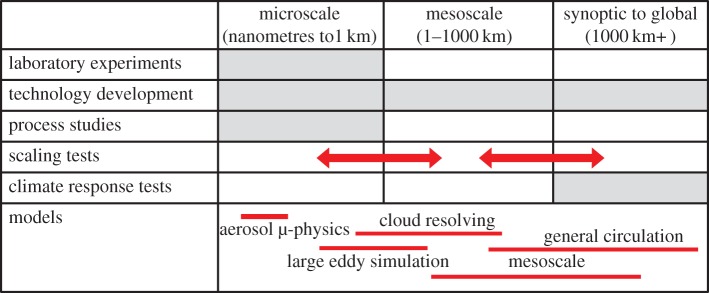
Mapping of experiment types and classes of models (red lines) to physical scales illustrates the breadth and complexity of solar geoengineering research. No single model or experiment can bridge the gap from smallest to largest scale. For example, microphysical models describe aerosol processes at scales of nanometres and cloud drops and ice crystals at micrometre to millimetre scale. Clouds (ranging from 10 to 1000 m) are addressed by large eddy simulation models and more generally by cloud resolving models. Mesoscale models and general circulation models (GCMs) have similar physics, but mesoscale models can be nested to provide high-resolution simulations that cannot be matched by GCMs. Chemistry can be built into dynamic models (typically mesoscale models and GCMs) or simulated in off-line chemical-transport models. The different types of field experiments, particularly process studies, scaling tests and climate response tests could bridge gaps between scales reducing the uncertainty of large-scale predictions of the risks and efficacy of SRM.

The concept of an atmospheric process is ambiguous, however, and reasonable definitions span a range of scales. Experiments that aim to test the fidelity of model predictions between various scales might therefore be much larger than the smallest scale process experiments. These distinctions between scales are illustrated in figure 1.

A common operational way to define *processes* is in terms of general circulation models (GCMs). Phenomena that take place at scales smaller than the model's numerical resolution are modelled by subgrid scale parametrizations, and one may define process experiments as those that aim to improve the fidelity of such parametrizations.

Today's high-resolution GCMs are discretized into grid boxes that are roughly 30–60 km in the horizontal and a few tenths of a kilometre in vertical extent in the lower atmosphere (about 50 levels each incorporating about 2% of the atmospheric pressure). The advection (flow) of energy and constituents—atmospheric chemical composition and aerosols—between grid boxes can be modelled with reasonable fidelity as can large-scale dynamical processes and atmospheric radiation. Uncertainties in subgrid scale processes are therefore among the most important uncertainties in predicting the risks and efficacy of SRM using GCMs. Put another way, if science had a perfect understanding of processes relevant to SRM at the grid-box scale, then uncertainty in global-scale predictions of SRM would be substantially reduced though they would not be eliminated.

Consider, for example, the possible loss of stratospheric ozone in response to aerosol injection. The large-scale ozone response depends first on the small-scale physical and chemical interactions that determine how the chemistry of an air parcel evolves, and second on the large-scale atmospheric dynamics that transport constituents within the stratosphere. Most (but not all) of the uncertainty in predicting the response of ozone to injection of a novel kind of aerosol stems from uncertainty in small-scale processes so it is possible for small-scale experiments to reduce uncertainty in large-scale predictive models.

Improved process models can reduce uncertainty, but they cannot eliminate it in most cases. The challenge of extrapolation from small to large scale, ‘up-scaling’, is harder in the troposphere. For marine cloud brightening (MCB), for example, the large-scale albedo response to the addition of cloud condensation nuclei (CCN) depends strongly on mesoscale processes—as when a change in radiative fluxes in response to CCN changes the cloud distribution in ways that produce larger changes in radiative flux than the initial perturbation. These mesoscale feedbacks may be a larger contribution to uncertainty than are the aerosol/cloud microphysics. Past field studies of cloud–aerosol–albedo interactions illustrate the difficulties in disentangling microphysical and macrophysical processes in these complex regimes [7]. This scale interdependence means that a range of field experiments that address specific atmospheric processes may be needed to bridge gaps between models (figure 1).

**Figure 2.**
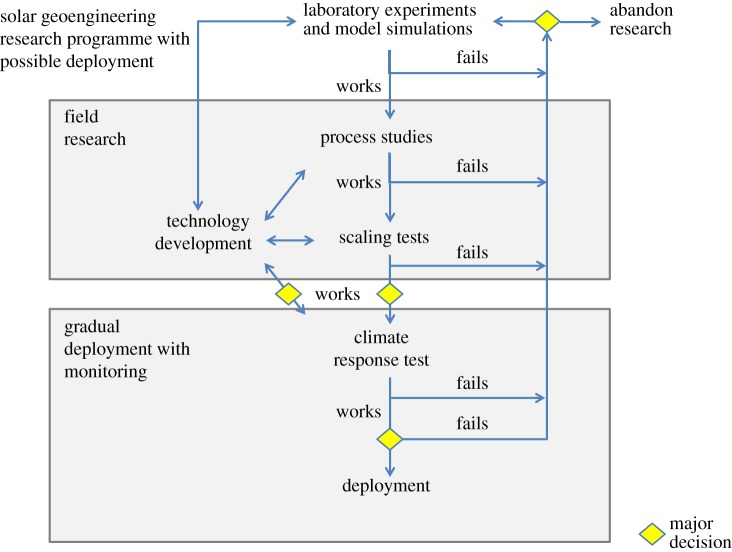
Schematic concept of a geoengineering research programme illustrating the phasing and interrelationship of various types of investigation defined in table 1. Incremental improvements in knowledge are linked to incremental increases in scale and risk. A decision to proceed from one stage to another should depend both on technical factors internal to the programme as well as on external factors such as the development of legitimate governance and evolving knowledge of the climate risks. The definition of ‘works’ and ‘fails’ is—of course—ambiguous and contingent. The distinction between *field research* and *gradual deployment with monitoring* represents a step-change in scale, risk and objectives, and should be made at a political level that transcends research management. Finally, even if the technology ‘works’ there may be good reasons to forgo deployment.

A research programme should be sequential and iterative as illustrated in figure 2, in the sense that one would not proceed to the next phase without a positive outcome from the prior phase. Determining that it is more difficult than expected to achieve desired outcomes, or that there are larger than expected undesired consequences would result in at least reconsideration and potentially a termination of any particular line of research.

With the exception of the last category—climate response tests—field experiments could be done with perturbations to radiative forcing that are negligible in comparison to the natural variability of climate at a global scale. Climate response tests are qualitatively different in that their aim is to produce a detectable climate response. Experiments at this scale should require a much different—more political, more international—decision process even for relatively small, incremental climate response tests. Indeed while the goals are partially scientific, a climate response test may not be meaningfully distinct from a gradual deployment. The goal of such a test/deployment would be to find unexpected problems before they become big enough to cause damage. (Of course, detection and attribution are limited by natural climate variability [6].) In a rational world, one would never try even a minimal climate response test unless (i) results from the other types of experiments in a sequential research programme (figure 2) suggested that benefits of geoengineering outweigh side effects and (ii) some form of international governance was established.

## 3. Workshop process

We convened a 1.5 day workshop at Harvard University in March 2014 that included a cross section of experimentalists, atmospheric scientists, engineers and social scientists (table 2). Experimentalists were asked to propose experiments for a hypothetical research programme for SRM field research. Proposers were asked to use the following template to prepare written summaries prior to the meeting:

— *Objectives and expected significance.* Clearly state the scientific goal(s) of the field experiment and address the following questions. What science question can this experiment answer? How does it address a major source of uncertainty for a given SRM method including efficacy and potential unintended consequences? What hypothesis will be tested? Why can the experiment objectives only be met via field test (e.g. what gap in model/simulation or laboratory test capability is being filled)? Is the experiment designed to produce a perturbation in radiative forcing at some level and if so, what is the rationale for the perturbation and what is the projected magnitude of the perturbation? Explain how these scientific objectives translate to specific, measurable quantities.
— *Technical approach and methodology.* Briefly describe how the experiment will be conducted including the location(s), duration, ‘actuation’ methods, instrumentation and observational platforms, models, data assimilation systems and any other technical infrastructure needs. Describe the analytical method including how the field test results can be disentangled from natural variability and other sources of ambiguity.
— *Risks and mitigations.* Enumerate likely and potential risks to operators, infrastructure, members of the public and ecosystems in the vicinity of the field test as well as the larger environment. Include biogeophysical and potential societal (including perceptual) issues that might arise in response to preparing for or conducting the experiment. Identify steps that might be taken to mitigate those risks.
— *Cost and schedule.* Provide a rough order of magnitude estimate for the experiment budget with key milestones indicating the major efforts involved in preparing, conducting and analysing the results of the experiment.

**Table 2.** Workshop attendees.

name	institution
Tom Ackerman	University of Washington
Jim Anderson	Harvard University
Neil Donahue	Carnegie Mellon University
Riley Duren	Jet Propulsion Laboratory
John Dykema	Harvard University
Sebastian Eastham	MIT
Steve Hamburg	Environmental Defense Fund
Josh Horton	Harvard University
David Keith	Harvard University
Frank Keutsch^a^	University of Wisconsin
Mark Lawrence^a^	IASS/Potsdam
Thomas Leisner^a^	KIT/Karlsruhe
Jane Long	Bipartisan Policy Center
Doug MacMartin	Caltech
David Mitchell^a^	Desert Research Institute
Granger Morgan	Carnegie Mellon University
Armand Neukermans	unaffiliated
Andrew Parker	Harvard University
Phil Rasch	Pacific Northwest National Laboratory
Lynn Russell^a^	Scripps
Stefan Schafer	IASS/Potsdam
Dan Schrag	Harvard University
Armin Sorooshian	University of Arizona
Graeme Stephens	Jet Propulsion Laboratory
Trude Storelvmo	Yale University
Matt Watson	University of Bristol
Debra Weinstein	Harvard University

^a^Attended only by phone or video.

At the workshop, eight distinct experiment proposals were presented. Reviewers (a subset of workshop participants) were then assigned to critique each proposal followed by plenary discussion. The reviews were discussed and summarized. Additionally, two breakout sessions were held to brainstorm other dimensions of field test experiments that were not addressed at this workshop but identified for potential future attention.

All the experiments were proposed by participants at the meeting. By agreement with participants we have not associated individual proposals with specific investigators because, while some proposals have been the subject of extensive and public prior analysis, we wanted to encourage participants to collaboratively develop new proposals and judged that assigning names would constrain discussion.

## 4. Summary and comparative analysis of experiments

The experiments proposed are summarized in §4*a*–*c* covering stratospheric aerosols, MCB and cirrus modification. We asked the proposers to comment on our summaries, but any remaining errors or inaccuracies are the responsibility of the authors. Finally, §4*d* covers the additional research concepts generated by meeting participants at the brainstorming sessions.

### (a) Marine cloud brightening

 *MCB experiments* are a phased sequence of tests; the summary here combines some information from two separate proposals that were presented during the workshop. One set of proposed experiments is described in Wood *et al*. [8]; a closely related suggestion that is combined with this here is a scale-up of the E-PEACE experiment [9]. These experiment proposals were informed by lessons learned from other atmospheric experiments including E-PEACE and VOCALS [10]. In addition, we include a larger scale test described in the literature [11] that was not presented or discussed at the workshop in order to illustrate further steps that might occur but are currently less well defined.

 *MCB phase 1* is technology development, to test the mechanism of evaporating droplets to form salt crystals and transporting the crystals upwards in the boundary layer under cloudless conditions over land. Salt nuclei would be generated by spraying seawater through small orifices; the purpose of this experiment is to ensure that appropriate size particles can be generated and lifted into the planetary boundary layer (rather than generating low hanging fog). Tests could evaluate the performance under different controlled stratification conditions; limitations in the ability to simulate complex boundary layer structure and dynamics motivate the need for well-designed outdoor tests.

 *MCB phases 2 and 3* would test the impact of salt particles as CCN in coastal marine boundary layer clouds; the intent of these phases is to develop and verify microphysical aerosol/cloud process understanding. Salt particles would again be lofted into the boundary layer to determine the impact on cloud properties; aircraft would be used to measure cloud and aerosol particle number and size distribution, cloud liquid water content and standard meteorological measurements. These experiments would first be conducted using land-based CCN injection (phase 2; shorter duration and smaller forcing), before moving to ocean-based injection (from a ship or buoy) in phase 3. In addition to the measurements of phase 2, the phase 3 test would include aircraft to measure cloud albedo. A key aspect of the experiment design is to predict the modifications to the cloud properties using large eddy simulation beforehand; insofar as the purpose is to ensure that the microphysics are understood, failure to predict results within reasonable variation of actual measurements constitutes failure in the experiment.

 *Mesoscale Ocean Cloud Experiment* (MOCX) was added by the authors after the workshop based on a proposed experiment by Latham *et al*. [11] to help complete the space–time–perturbation phase space of this study. MOCX would test the impact on cloud albedo responses at larger scale and over longer duration, to determine albedo response to aerosol perturbations of different strengths as a function of environmental (meteorology, thermodynamic) and cloud macrophysical conditions (e.g. liquid water path, cloud thickness). This scaling test inherently requires longer durations and/or areas than MCB phases 2 and 3 in order to obtain data over a larger range of cloud conditions, and to include measurement of any compensation effects on clouds outside of the modified area. Such a test would clearly only occur after successful results from earlier phases. The estimated local forcing, duration and spatial extent of this test shown in table 3 are taken from [11] but clearly until earlier phases were conducted, there is greater uncertainty about the appropriate scale.

**Table 3.** Summary of field test experiment concepts explored in this study. For each experiment, we provide (if known) the local peak radiative forcing (ΔRF), area of the experiment domain (*A*), individual test duration (*T*), number of tests in an experiment (*N*), equivalent energy (*E*=*pΔ*RF×*A*×*T*×*N*), the primary composition and mass of materials injected into the atmosphere, and the type of experiment. TBD, to be determined. Experiment costs are very uncertain. In each case, experiment duration is limited to the active period of injection (in some but not all cases, continuous) and does not indicate months of preparatory efforts or data analysis. ΔRF represents the maximum quasi-instantaneous change in radiative forcing over the domain indicated in response to a given experiment (assuming the experiment is operating at ‘steady state’); it does not account for natural variability or start-up.

exp. no.	informal title	category type(s)	cost ($M)	local forcing, area, duration and equivalent energy	material and mass	synopsis
1	SCoPEx	process study	10	ΔRF=0.01–0.1 Wm^−2^	10^3^ g of S and less than 10^5^ g of H_2_O	stratospheric propelled balloon to test chemistry response to H_2_SO_4_ and H_2_O and to test aerosol microphysical models
				*A*=10^1^ km^2^		
				*T*=1 week		
				*N*=4		
				*E*=2.4×10^12^ J		
2	cirrus cloud seeding	process study	0.5	ΔRF=1–10 Wm^−2^	3×10 g of BiI_3_	ice nucleation seeding from aircraft in upper troposphere to test cirrus dispersal mechanisms
				*A*=10^2^ km^2^		
				*T*=1 week		
				*N*=4		
				*E*=2.4×10^15^ J		
3	MCB phases 1–2	technology development, process study	1	ΔRF=0.1–5 Wm^−2^	sea salt	(i) boundary layer injection of sea salt from coastal site to test sprayer technology; (ii) coastal test of cloud brightening
				*A*=10^2^ km^2^		
				*T*=2 weeks		
				*N*=4		
				*E*=2.4×10^15^ J		
4	MCB phase 3	process study, scaling test	2	ΔRF=5–50 Wm^−2^	sea salt	ocean test of MCB (sea salt injection into boundary layer from single ship—e.g. single enhanced ship track)
				*A*=10^2^ km^2^		
				*T*=4 weeks		
				*N*=4		
				*E*=4.8×10^16^ J		
5	MSGX	scaling test, technology development	100	ΔRF=0.2 Wm^−2^	5×10^8^ g of S	sustained stratospheric injection of H_2_SO_4_ from aircraft, observe mesoscale effects from satellites and aircraft
				*A*=10^6^ km^2^		
				*T*=6 months		
				*N*=1		
				*E*=1.3×10^19^ J		
6	climate response test	climate response test	>1000	ΔRF=0.5 Wm^−2^	1×10^12^ g of S per year	test global climate response to large-scale modulated input (either stratospheric sulfate or MCB)
				*A*=5×10^8^ km^2^		
				*T*=10 years		
				*N*=1		
				*E*=8×10^22^ J		
7	MOCX	scaling test, technology development	10	ΔRF=50–100 Wm^−2^	sea salt	large-scale test of MCB in open ocean with multiple, coordinated ships
				*A*=4×10^4^ km^2^		
				*T*=4 weeks		
				*N*=4		
				*E*=7.7×10^19^ J		
8	SPICE-2	technology development	0.5	ΔRF=none	10^3^ g of H_2_O	test 1 km scale balloon injection approach
				*A*=10^1^ km^2^		
				*T*=2 weeks		
				*E*=none		
9	volcanogenic particles	process study	2	ΔRF=none	small amounts of H_2_S, SO_2_, 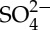 , SiO_2_	observe physical/chemical fate of candidate particles from (i) volcano and (ii) aircraft injection (S-bearing species and SiO_2_)
				*A*=TBD km^2^		
				*T*=TBD days		
				*E*=TBD		

There is residual uncertainty in key quantitative properties for all of the MCB experiments described here. For example, while they each involve spraying of natural sea salt, the exact salt mass flux for a given experiment is still very uncertain in part because even when the amount of CCN is known the mass of salt depends on the particle size and thus on the spray technology used. Additionally, the ΔRF numbers shown for these experiments in table 3 represent the estimated peak, quasi-instantaneous local perturbation over the indicated area of the experiment. For example, a local ΔRF of 100 W m^−2^ (consistent with forcing from natural clouds) is not out of the question over some areas for some period of time but the average ΔRF over the experiment will presumably be considerably smaller. There is considerable flexibility in the range of target local ΔRF and hence we illustrate both the low- and high-range cases.

### (b) Stratospheric experiments

 *The Stratospheric Controlled Perturbation Experiment* (SCoPEx) [12] comprises (i) a *stratospheric* balloon and propelled gondola that can reenter an experimental volume many times during a diurnal cycle, (ii) the ability to generate controlled perturbations in water vapor and aerosols, and (iii) an *experimental* design that measures responses to perturbations—such as the coagulation of aerosols and their role in activation of chlorine by heterogeneous reactions—and allows quantitative test of models. A balloon was selected over an aircraft because the long duration and low flight velocity allow repeated sampling of an air mass. Initial analysis suggests that the science objective can be achieved with total perturbation less than 1 kg S and less than 100 kg H_2_O. Science requirements are satisfied by selecting cold conditions in the mid-latitude lower stratosphere; initial plans call for operating from a continental US site at altitudes of approximately 20 km during periods of low winds that occur in May and September.

 *Possible extensions*. If the platform, fight operations and data analysis for SCoPEx were proved, then later follow-on experiments might include the following.

— Longer duration operations (e.g. 10 days) to enable more extensive test of the coevolution of aerosols and chemistry. The super-pressure balloon proposed for SCoPEx has been previously demonstrated with flight durations exceeding 30 days, and preliminary investigations suggest that mission durations of the order of 10 days might be feasible given a moderate increase in the vehicle's sustained speed.
— Dispersion or generation of engineered particles and measurement of their impact on local atmospheric chemistry.

 *Mesoscale Stratospheric Geoengineering Experiment* (*MSGX*) aims to test sulfate aerosol geoengineering at a scale sufficiently large to enable quantitative tests of stratospheric mixing, aerosol dynamics, the impact of aerosol heating rates on dynamics, ozone chemistry and radiative forcing, and to enable comparison of *in situ* and remote sensing observations. These requirements might be met by a perturbation with a horizontal length scale of 1000 km in each dimension and a vertical scale of a few kilometres. Such a perturbation might be achieved in a few days with approximately five aircraft with specifications roughly equivalent to the re-engined G650 studied by McLellan *et al*. [13]. Total sulfate mass: approximately 500 t S; peak patch-average radiative forcing: approximately 0.2 W m^−2^.

### (c) Cirrus cloud modification

While not strictly speaking solar (short-wave) radiation management, cirrus cloud cover might be artificially reduced by seeding with efficient ice nuclei (IN) [14,15] such as bismuth tri-iodide (BiI_3_); reduced cirrus cover would increase outgoing long-wave radiation. Typically, IN are rare, leading to mostly homogeneous rather than heterogeneous nucleation; increasing IN may deplete water vapour yielding fewer ice crystals. An initial field test would aim at process understanding of cloud microphysics through small-scale seeding to probe processes with fidelity not attainable with indoor cloud chambers. There is some uncertainty on how large a seeding area would be required (due to uncertain mixing over the time scales involved) but an initial estimate is over a 10×10 *km* region using 30 g of BiI_3_ over a period of weeks. Aircraft would be used to monitor IN distribution and cirrus cover in both seeded and control areas. The test would be done at high latitudes where the relative benefit from long-wave changes versus offsetting short-wave changes is highest [16].

### (d) Other

A number of additional field research activities were identified during a brainstorming session at the workshop but were not explored in detail. The concepts can be divided into three broad categories as follows:

— tests associated with other means of altering radiative fluxes such as increasing the albedo of the ocean surface (e.g. micro-bubbles) or cirrus thinning via aerosol or black carbon injection,
— tests associated with interventions in regional climate or weather such as attempts to reduce the severity of heat waves [17], and
— tests aimed at assessing risks and impact of any approach, particularly impacts on surface processes (terrestrial and ocean ecosystems, solar power), tropospheric chemistry, cirrus generation (from fall-out of stratospheric aerosols), stratification, water vapour transport, turbulence or other concerns.

## 5. Discussion

Participants at the March 2014 workshop attempted to describe a broadly representative portfolio of field experiments for solar geoengineering. The workshop and this paper have several limitations. First, while the experiments described here capture the ideas of a number of people who have been thinking deeply about this problem, this is a ‘bottom-up’ collection of individual projects rather than a systematic ‘top-down’ plan based on first articulating systematic research priorities and then identifying what experiments (if any) would be required to address these.

Second, most of the experiments proposed did not include provisions to measure and attribute the impact of the experiment on radiative forcing. This is an important caveat given the ultimate aim of proposed solar geoengineering methods. This is not to suggest that process studies of chemistry, dynamical responses, etc., are less important than direct measurement of radiative forcing but merely to point out the challenge in drawing inference about large-scale changes in radiative forcing from relatively small perturbative experiments.

Third, not all of the experiments gave a strong argument for why a perturbative field test is warranted rather than a laboratory experiment (e.g. large cloud chamber) or passive process studies (e.g. better observations of natural interactions between IN and cirrus) although this was perhaps due more to the limitations of our brief study than an inability to provide such rationale. It is likely that passive process studies are needed to provide the background (or control experiment) either prior to or during an active field experiment in order to support attribution. It is equally possible that passive studies alone may not provide sufficient reduction in uncertainty to predict geoengineering outcomes with acceptable levels of confidence—particularly for addressing scaling issues [18]. In some cases, arguments for more incremental reductions in uncertainty through passive studies in lieu of perturbative experiments could delay or complicate robust decision-making. We suggest that an appropriate scientific criterion is that research that provides the largest reduction in uncertainty for a given research investment and risk should receive priority, whether they be passive studies or active field experiments.

With these caveats in mind, we make the following observations about the characteristics of this portfolio of SRM field experiments and about its implications for assessments of governance, research priorities and observational needs.

**Figure 3.**
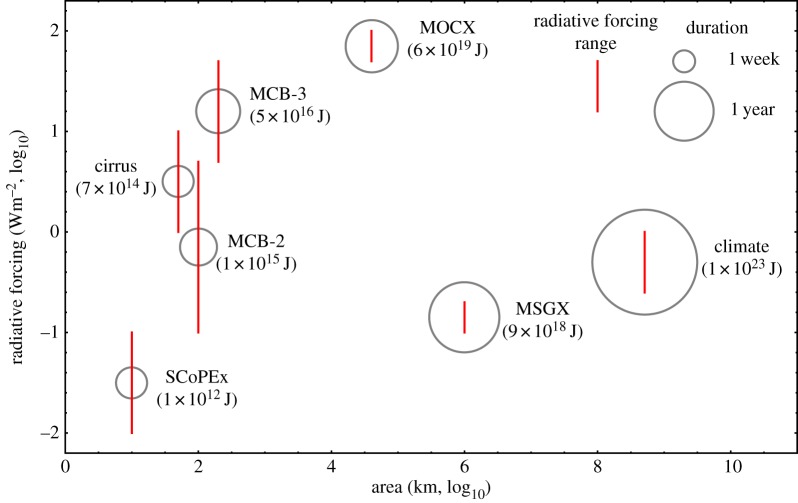
Comparison of the climate forcing of field experiments. Area and local radiative forcing (ΔRF) are plotted as red bars on the axes of a log–log plot, where the bars indicate the range of possible ΔRF from table 3. Duration is indicated by the size of the grey circles as show in the key (the area of the circles is proportional to the square root of the duration). A useful measure of the total climate forcing is the product area×duration×ΔRF which has units of energy; this value is given under the experiment name (using average of the maximum and minimum ΔRF). The aggregate forcing energies span 11 orders of magnitude. Finally, note that the cirrus, MCB-3 and MCB-2 all have an area of 100 km^2^, but the *x*-axis values have been offset in the figure to show the three red range bars.

Figure 3 provides a quantitative comparison of the area, radiative forcing and duration of the first seven experiments listed in table 3. A log–log plot is required given the very large range of parameters in the portfolio. For example, the cirrus experiment is 50 times shorter in duration than MSGX. The area of the SCoPEx domain is 100 000 times smaller than that of MSGX.

No single metric captures the aggregate climate impact of the proposed experiments. For global climate impacts, the perturbation energy (area×duration×ΔRF) provides a reasonable measure of the total forcing. It is useful to compare the perturbation energy of the experiments to the climate's inter-annual variability in global reflected solar flux which is about 0.2 W m^−2^ [19] corresponding to a perturbation energy of 3×10^22^ J. The climate experiment, for example, has a perturbation energy three times larger than this measure of natural variability while SCoPEx is a factor of 3×10^10^ smaller.

While the perturbation energies give some measure of the global forcing they tell us nothing about local forcing and impacts. One plausible measure of local impacts is the product of ΔRF and duration. On this measure, the MCB experiments have much larger impacts than do experiments related to cirrus modification or stratospheric aerosols.

Solar geoengineering field experiments involve processes that span the Earth system including domains with very different controlling processes and sources of uncertainty. For example, many experiments involving stratospheric processes may be more appropriately focused on aerosol microphysics and chemistry rather than mesoscale atmospheric mixing, whereas experiments involving boundary layer clouds will likely need to account equally for microscale aerosol–cloud interactions as well as mesoscale mixing. These differences arise because in the marine boundary layer local changes in radiative forcing due to aerosol addition will have large and fast-acting changes on local transport, whereas for the density of stratospheric aerosols relevant for SRM the impact of local radiative forcing on the local dynamics is much less important. In summary, participants in our workshop see greater challenges in extrapolating results across scales (see the ‘scaling tests’ in table 1 and the depiction of model scales figure 1) for MCB than for stratospheric aerosols. These differences in atmospheric physics demonstrate the difficulty of comparing experiments across domains because even when experiments have equivalent size, duration and radiative forcing they may have dramatically different implementation modes, costs, risks and scientific merit.

There may be potentially strong co-benefits to climate science for some solar geoengineering field tests. For example, some of the experiments targeting MCB could also reduce uncertainty in the cloud–aerosol indirect effect which remains a major source of uncertainty in climate models with significant implications on improved fidelity for projections, impact assessments and adaptation planning. It could be that controlled release experiments that perturb marine clouds or stratospheric aerosols or cirrus clouds provide greater reductions in uncertainty for key processes than that offered by passive studies. This consideration should be factored into assessments of whether a given project warrants a perturbative field experiment. However, the research community should also be mindful to ensure that transparency of research motivations is maintained; that is—do not obscure the true intent of an experiment with ‘dual-badging’.

Workshop participants highlighted the need for an end-to-end research roadmap for solar geoengineering that would articulate the sequencing and dependencies of a research programme that recognizes and includes: technology development, process studies, scaling tests, and potentially, climate response tests (figure 1 and table 1). Future assessments of proposed geoengineering field experiments should consider all of these categories to help address the question: what problem are we trying to solve? Such a roadmap could be useful in informing decisions about research programme scope and where to assign responsibility for the different elements—including potential alignment with existing research programmes. Finally, such a roadmap must articulate the distinctions between scales, goals and risks of proposed experiments and provide a framework for making decisions about further progress or abandonment as shown in figure 2.

Beyond the technical specifics, we draw three observations about the portfolio as a whole that may be relevant to readers concerned with public policy and governance. First, the very existence of a *portfolio* indicates that there is a growing set of potential SRM field experiments being developed by researchers with a track record of atmospheric science field experiments. Second, the range of plausible experiments is very broad, spanning many orders of magnitude in measures such as physical scale, duration, total climate forcing and risk. Third, many of the experiments involve perturbations that are small compared with the physically similar perturbations due to commonplace industrial activities such as single flights of a commercial jet aircraft or single ocean crossings of a large bulk transport ship.

Collectively, these three observations imply that public policy (or governance) of SRM field experiments cannot sensibly make unitary assumptions about the scale and risk of such experiments. Rather, they must make distinctions between experiments that grapple with the actual diversity of scale and risk.

In closing, we have engaged a group of leading researchers to craft a hypothetical field experiment portfolio for solar geoengineering to help inform assessments of research priorities, governance considerations and observational needs. The examples described here are representative rather than comprehensive. By offering a notional portfolio that spans a broad space of experiment spatial area, duration, radiative forcing and equivalent energy, we offer quantitative examples to support critical discussion. More importantly, we explore basic framing issues including definition of key experiment categories, physical scales and other considerations to include in future assessments of this important topic.
